# Synergistic and Antagonistic Mutation Responses of Human MCL-5 Cells to Mixtures of Benzo[*a*]pyrene and 2-Amino-1-Methyl-6-Phenylimidazo[4,5-*b*]pyridine: Dose-Related Variation in the Joint Effects of Common Dietary Carcinogens

**DOI:** 10.1289/ehp.1409557

**Published:** 2015-06-19

**Authors:** Rhiannon David, Timothy Ebbels, Nigel Gooderham

**Affiliations:** Computational and Systems Medicine, Imperial College London, London, United Kingdom

## Abstract

**Background:**

Chemical carcinogens such as benzo[*a*]pyrene (BaP) and 2-amino-1-methyl-6-phenylimidazo[4,5-*b*]pyridine (PhIP) may contribute to the etiology of human diet-associated cancer. Individually, these compounds are genotoxic, but the consequences of exposure to mixtures of these chemicals have not been systematically examined.

**Objectives:**

We determined the mutagenic response to mixtures of BaP and PhIP at concentrations relevant to human exposure (micromolar to subnanomolar).

**Methods:**

Human MCL-5 cells (metabolically competent) were exposed to BaP or PhIP individually or in mixtures. Mutagenicity was assessed at the thymidine kinase (TK) locus, CYP1A activity was determined by ethoxyresorufin-*O*-deethylase (EROD) activity and qRT-PCR, and cell cycle was measured by flow cytometry.

**Results:**

Mixtures of BaP and PhIP produced dose responses different from those of the individual chemicals; we observed remarkably increased mutant frequency (MF) at lower concentrations of the mixtures (not mutagenic individually), and decreased MF at higher concentrations of the mixtures, than the calculated predicted additive MF of the individual chemicals. EROD activity and *CYP1A1* mRNA levels were correlated with TK MF, supporting involvement of the CYP1A family in mutation. Moreover, a cell cycle G2/M phase block was observed at high-dose combinations, consistent with DNA damage sensing and repair.

**Conclusions:**

Mixtures of these genotoxic chemicals produced mutation responses that differed from those expected for the additive effects of the individual chemicals. The increase in MF for certain combinations of chemicals at low concentrations that were not genotoxic for the individual chemicals, as well as the nonmonotonic dose response, may be important for understanding the mutagenic potential of food and the etiology of diet-associated cancers.

**Citation:**

David R, Ebbels T, Gooderham N. 2016. Synergistic and antagonistic mutation responses of human MCL-5 cells to mixtures of benzo[*a*]pyrene and 2-amino-1-methyl-6-phenylimidazo[4,5-*b*]pyridine: dose-related variation in the joint effects of common dietary carcinogens. Environ Health Perspect 124:88–96; http://dx.doi.org/10.1289/ehp.1409557

## Introduction

Consumption of red meat is positively correlated with some human cancers, and cooking meat produces heterocyclic amines (HCAs) and polycyclic aromatic hydrocarbons (PAHs) (Sinha et al. 2005). The HCA 2-amino-1-methyl-6-phenylimidazo[4,5-*b*]pyridine (PhIP) is bioavailable to humans who consume cooked meat (ingesting 0.1–15 μg PhIP per day) (Felton et al. 2002). PhIP is a rodent carcinogen (Sugimura 1997), inducing cancer in the prostate, colon, and mammary gland of rats (Crofts et al. 1997; Ito et al. 1991). The activation of PhIP to DNA-damaging species occurs via *N*-hydroxylation catalyzed by cytochromes P450 (CYP) 1A1 and 1A2 (Crofts et al. 1997; Zhao et al. 1994), thereby forming promutagenic adducts at C8 of guanine and resulting in GC:TA transversions and deletions (Boyce et al. 2014; Lynch et al. 1998). Benzo[*a*]pyrene (BaP) is a carcinogen that is generated by incomplete combustion of organic substances, leading to the contamination of numerous foodstuffs (Lijinski and Shubik 1964). BaP is metabolized by enzymes from the CYP1A family to epoxide derivatives that form DNA adducts and result in mutation and tumors [International Agency for Research on Cancer (IARC) 2010]. Through the consumption of contaminated food, the average human daily exposure to BaP is estimated to be 1–500 ng (IARC 2010). Experimental studies suggest a positive link between exposure to BaP and cancer in animals and in humans (Sinha et al. 2005).

Published assessment of genotoxic carcinogens, particularly dietary carcinogens, in mixtures is limited. Current approaches to mixtures risk assessment include whole-mixture– and component-based methods [Agency for Toxic Substances and Disease Registry (ATSDR) 2004; U.S. Environmental Protection Agency (EPA) 2000]; whole-mixture approaches are preferred because they account for unidentified components and interactions between chemicals. However, the complexity and variability of mixtures makes this approach difficult, and component-based methods such as dose or response additivity are often used (Boobis et al. 2011; COT 2002; Lutz et al. 2005). Synergistic effects from interactions between PAHs on DNA adduct levels have been reported (Staal et al. 2007; Tarantini et al. 2009, 2011), and prolonged activation of DNA damage signaling suggestive of persistent DNA damage by mixtures of PAHs has been observed (Jarvis et al. 2013; Mattsson et al. 2009; Niziolek-Kierecka et al. 2012), suggesting that the present risk assessment strategies may underestimate risk. Contrasting studies showing antagonistic or additive effects from mixtures of PAHs (Courter et al. 2008; Mahadevan et al. 2007; Marston et al. 2001; Staal et al. 2008; White 2002) or of heterocyclic aromatic amines (Dumont et al. 2010) have been published. To our knowledge, there is no information regarding mixtures of PAHs with HCAs at concentrations that are relevant to human exposure [micromolar to subnanomolar concentrations, with the highest concentrations in gastrointestinal (GI) microenvironments]. Such information is important for risk assessment of food-borne chemical carcinogens. Thus, our aim was to determine the mutagenic response to mixtures of BaP and PhIP at concentrations relevant to human exposure.

## Methods

*Materials.* RPMI-1640 medium (with phenol red, without L-glutamine and histidine), heat-inactivated horse serum (HIHS), L-glutamine, penicillin/streptomycin, and hygromycin B were obtained from Life Technologies (Paisley, UK). All other chemicals were purchased from Sigma-Aldrich (Poole, UK).

*Cell culture.* MCL-5 is a human B lymphoblastoid cell line that constitutively expresses CYP1A1 (Crespi et al. 1991) and stably expresses transfected CYP3A4, CYP2E1, CYP1A2, CYP2A6, and microsomal epoxide hydrolase (Crespi et al. 1991). Thus, this cell line can activate BaP and PhIP to DNA-damaging species without the need for exogenous activation systems (S9 fraction). Moreover, these cells are relevant to the human exposure route (the diet) because CYP1A1 is expressed in the GI tract (Paine et al. 2006). Cells were cultured in RPMI-1640 medium supplemented with 10% (v/v) HIHS, 2 mM L-glutamine, 100 U/mL penicillin, 100 μg/mL streptomycin, 2 mM histidinol, and 200 μg/mL hygromycin B; this medium was called R10. Stocks were not cultured beyond 5 weeks (Johnson et al. 2010).

*HAT treatment of cells.* To remove background mutants, MCL-5 cells were cultured for 3 days in R10 containing HAT [hypoxanthine, aminopterin, thymidine; Hybri-Max™ (Sigma-Aldrich, Poole, UK)], which is lethal to cells harboring mutations at the TK locus (Busby et al. 1994). Subsequently, the cells were transferred to media containing HT (hypoxanthine, thymidine; Hybri-Max™) for 24 hr; then, the mutant-depleted cultures were maintained for 4 days in normal media prior to freezing.

*TK forward mutation assay.* Mutation assays used HAT-treated cells (50 mL at 4 × 10^5^ cells/mL) with BaP or PhIP or binary mixtures to achieve the final concentrations outlined in Table 1. Dimethylsulfoxide (DMSO; 0.1% vol/vol) and ethyl methanesulfonate (EMS; 10 μg/mL) were the negative and positive controls for all experiments, respectively. Mutation data were considered acceptable provided that the relative total growth (RTG) and mutant frequency (MF) for both DMSO and EMS controls complied with historical data and that RTG additionally complied with Organisation for Economic Co-operation and Development (OECD) guidelines (data not shown) (OECD 1997). Published methodology (Clements 2000) was followed, with some optimizations. Cells were treated for 24 hr at 37°C and 5% CO_2_ in RPMI-1640 containing all supplements but with reduced serum [5% (v/v) HIHS]. After treatment, the cellular concentration was adjusted to 4 × 10^5^ cells/mL, and the cells were subcultured daily for 2 additional days to determine the relative suspension growth (RSG). On the third day, cells were plated (10 cells/well in 2 × 96-well plates) for cloning efficiency (CE) and 20,000 cells/well in 3 × 96-well plates in trifluorothymidine (TFT; 4 μg/mL) to determine thymidine kinase (TK) MF. Plates were incubated for 21 days to determine MF. RTG was calculated to estimate cytotoxicity, and MF was expressed as mutants/10^6^ viable cells (Clements 2000).

**Table 1:** Relative total growth (RTG) and background-corrected mutation frequency (MF/106 viable cells) at the thymidine kinase (TK) locus following treatment with different concentrations of BaP and PhIP alone or in selected combinations.

Treatment and concentration (M)	RTG^*a*^	TK MF^*b*^
BaP		0.1 ± 1.3
10^–10^	98.7 ± 0.7	0.1 ± 1.5
10^–9^	95.1 ± 3.8	0.1 ± 1.8
10^–8^	87.6 ± 5.9	1.3 ± 1.2
10^–7^	85.6 ± 6.1	30.1 ± 4.3**
2.5 × 10^–7^	85.6 ± 7.9	44.5 ± 4.7***
7.5 × 10^–7^	74.1 ± 1.4	46.5 ± 6.9***
10^–6^	74.2 ± 4.0	54.4 ± 3.6***
2.5 × 10^–6^	85.9 ± 6.5	61.4 ± 8.5***
7.5 × 10^–6^	64.4 ± 0.4	76.9 ± 10.5***
10^–5^	56.4 ± 7.7	0.1 ± 1.3
PhIP
10^–9^	98.0 ± 3.9	0.7 ± 0.4
10^–8^	113.2 ± 3.2	0.1 ± 1.3
10^–7^	112.6 ± 6.8	5.6 ± 2.4
10^–6^	114.2 ± 5.9	2.1 ± 1.6
10^–5^	103.9 ± 2.9	5.1 ± 1.5
5 × 10^–5^	89.2 ± 4.9	7.0 ± 1.6**
7.5 × 10^–5^	78.6 ± 4.7	8.8 ± 1.2*
10^–4^	83.0 ± 5.4	7.9 ± 2.4**
BaP + PhIP
10^–9^ + 10^–9^	109.8 ± 8.6	2.47 ± 2.13
10^–7^ + 10^–6^	95.2 ± 7.8	43.6 ± 11.38***
10^–7^ + 5 × 10^–5^	113.2 ± 12.2	0.1 ± 4.72
10^–7^ + 10^–4^	89.4 ± 9.4	4.28 ± 2.81
2.5 × 10^–7^ + 10^–6^	117.5 ± 8.8	56.81 ± 10.68***
10^–6^ + 10^–6^	118.2 ± 1.2	39.71 ± 4.56*
10^–6^ + 5 × 10^–5^	60.1 ± 8.2	17.5 ± 4.17*
10^–6^ + 10^–4^	113.7 ± 13.3	2.93 ± 3.14
10^–5^ + 10^–6^	48.3 ± 3.4	41.1 ± 5.22***
10^–5^ + 5 × 10^–5^	100.9 ± 7.4	16.85 ± 5.28*
10^–5^ + 10^–4^	81.6 ± 12.5	7.92 ± 2.49
DMSO negative control average range, 4.9–18.6 MF/10^6^ viable cells; EMS positive control average range, 74.6–125.5 MF/10^6^ viable cells. Historical controls: TK: DMSO, 13.3 ± 9.4 MF/10^6^ viable cells ± standard deviation; EMS (positive control), 99.4 ± 40.4 MF/10^6^ viable cells ± standard deviation. ^***a***^RTG values are percent means ± standard error of the mean (SEM), *n *= 3–12. ^***b***^Data are presented as background-corrected means ± standard error of the mean (SEM), *n *= 3–12. Significance compared with the DMSO control calculated using one-way analysis of variance (ANOVA) with Dunnett’s post-test (*p ≤ 0.05, **p ≤ 0.01, ***p ≤ 0.001).		

*Ethoxyresorufin-*O*-deethylase (EROD)*. Ethoxyresorufin-*O*-deethylase (EROD, an indicator of CYP1A activity) was measured as the conversion of 7-ethoxyresorufin (7-ER) to resorufin. Cell suspensions (10 mL at 4 × 10^5^ cells/mL) were treated with selected concentrations of BaP, PhIP, or combinations of BaP and PhIP for 24 hr.Then, 3 × 10^6^ cells were collected for EROD activity analysis by centrifugation [200 × *g*, 5 min, room temperature (RT)], washed once in phenol red–free/serum-free RPMI-1640 media (R0) and resuspended in 1 mL R0 media in 24-well plates. We added (1 μL DMSO to give a final concentration of 8 μM) 7-ER to the wells, and the cells were incubated for 90 min at 37°C. Fluorescence was measured at λexcitation = 560 nm and λemission = 590 nm every 10 min (POLARstar Galaxy Microplate Reader, BMG Lab Technologies). Activity was expressed as picomoles of resorufin produced per minute per 10^6^ cells using a resorufin standard curve.

*Protein determination.* Cells (3 × 10^6^) collected by centrifugation (450 × *g*, 5 min, RT) were treated with RIPA buffer (Sigma) containing Halt protease inhibitor cocktail (Life Technologies) for 30 min on ice. The lysate was clarified by centrifugation (8,000 × *g*, 10 min, 4°C), and the supernatant was collected and stored at –20°C. Lysate protein was determined using the bicinchoninic acid (BCA) assay (Pierce, Thermo Scientific) following the manufacturer’s instructions.

*Immunoblotting.* Protein samples (20 μg) were mixed with 5× loading buffer containing β-mercaptoethanol, and the volume was adjusted to 25 μL. Samples were boiled (95°C, 5 min), centrifuged (10,000 × *g*, 5 min), and loaded onto a 10% sodium dodecyl sulfate (SDS)-polyacrylamide gel and electrophoresed (100 V, 1.5 hr). Proteins were transferred to nitrocellulose membrane (150 V, 400 mA, 1.5 hr), and Ponceau S stain was used to confirm the transfer. The membrane was blocked (PBS-T 0.1% Tween 20, 5% milk powder) and then incubated with antibodies for MSH2 or MSH6 (Abcam, 1:1,000 dilution, 4°C overnight). The membrane was incubated with a horseradish peroxidase (HRP)–coupled goat anti-rabbit or goat anti-mouse secondary antibody (Abcam, 1:10,000 dilution) for 1 hr at room temperature. Protein bands were detected using Luminata Forte Western HRP substrate (Merck-Millipore) and visualized using the ChemiDoc XRS+ Molecular Imager System (BIO-RAD, San Francisco, CA, USA). Blots were probed for β-actin as a loading control; the primary antibody (1:200; Sigma) was incubated for 1 hr; then, the secondary antibody (goat anti-mouse, 1:10,000; Abcam) was incubated for 1 hr.

*RNA extraction and quantitative RT-PCR (qRT-PCR).* Following treatment with selected concentrations of mixtures of BaP and PhIP, cells (3 × 10^6^) were collected by centrifugation (200 × *g*, 5 min, RT). The pellets were resuspended in 0.5 mL TRIzol (Invitrogen, Paisley, UK) for RNA extraction, quantified (Implen nanophotometer; GmbH, Munchen, Germany), and the A260/280 and A260/230 ratios were used to assess RNA quality. To synthesize cDNA, 1 μL of random primers was added to 500 ng of RNA (adjusted to a final volume of 15 μL with RNase/DNase–free dH_2_O) and incubated for 5 min at 65°C. The mixture was placed on ice before adding 0.2 mM dNTPs, 5 μL 5× first-strand buffer, 2 μL 0.1 mM DTT, and 0.5 μL Superscript II reverse transcriptase (Superscript II kit, Life Technologies). Samples were run on a thermocycler (25°C, 10 min; 42°C, 90 min; 70°C, 15 min). CYP1A1 cDNA was amplified by qRT-PCR. As an internal control, endogenous glyceraldehyde-3-phosphate dehydrogenase (GAPDH) cDNA from the same cellular extracts was also amplified. cDNA was amplified using a Taqman Fast 2× Universal PCR master mix, No AmpErase UNG kit (Life Technologies); all samples were analyzed in triplicate. qRT-PCR data were analyzed using the ABI 7500 Sequence Detection System (Life Technologies) and the comparative C_T_ (threshold cycle) method (ΔC_T_ method) (Livak and Schmittgen 2001). Calibration was based on the expression of GAPDH.

*Flow cytometry analysis of cell cycle distribution.* Cell cycle stage was determined using flow cytometry. MCL-5 cells were resuspended in 1 mL 70% ethanol (in phosphate-buffered saline; PBS) at –20°C. Cells were collected by centrifugation (450 × *g*, 10 min, 4°C), washed once in PBS, resuspended in 200 μL PBS containing propidium iodide (5 μg/mL) and RNase A (0.1 mg/mL), incubated (37°C, 30 min), and examined by flow cytometry (Fortessa II, Beckman Coulter); cell cycle distribution was determined using FloJo software (Tree Star Inc., Ashland, OR, USA).

*Statistical analysis of mutation data to determine synergy/antagonism.* Median effect plot and combination index (CI). Data were analyzed using the method of Chou (2006) with background-corrected MF. Median effect plots of log(dose) versus log(f_a_/f_u_), where f_a_ is the fraction affected (MF/10^6^ viable cells) and f_u_ is 10^6^ – f_a_, were drawn for individual chemicals to obtain the slope (m), the median effect dose (Dm, calculated as the antilog of the x intercept when y = 0), and the Pearson correlation coefficient (*r*), which signify the shape of the dose–effect curve, the potency (IC_50_), and the conformity of the data to the mass action law, respectively. From these plots, doses of the individual chemicals required to produce the mixture effect were calculated using Equation 1:

Dx = Dm[f_a_(mix)/1 – f_a_(mix)]^1/m^. [1]

The CI was calculated using Equation 2:

CI = D_1_/Dx_1_ + D_2_/Dx_2_, [2]

where D_1_ and D_2_ are the concentrations of the individual chemicals used in the mixture, and subscripts 1 and 2 refer to the two components of the mixture.

For the CI calculation, the value of D was also calculated using Equation 1. Whereas D represents the dose of individual chemical used in the mixture, Chou (2006) states that “dose and the effect are interchangeable since the dose (D) for any given effect (f_a_) can be determined if the values for Dm and m are known.” Because Dm and m were obtained from the median effect plot, from which the Dx values were also derived, it was noted that calculating D based on these values gave modified doses; thus, we have adjusted D to reflect the median effect plot.

Synergism and antagonism are determined from CI and are subdivided into nearly additive (NDAd, 0.9–1.1), moderate synergism/antagonism (mS, 0.7–0.90/mA, 1.1–1.45), synergism/antagonism (S, 0.3–0.7/A, 1.45–3.3), and strong synergism/antagonism (sS, < 0.3/sA, > 3.3) (Chou 2006).

*Interaction factor (IF).* Data were also analysed using the interaction factor (IF), which was calculated with background-corrected MF following the method described by Danesi et al. (2012). The IFs were calculated using Equation 3:

IF = G_1_G_2_ – G_1_ – G_2_ + C, [3]

where G_1_G_2_ is the MF obtained for treatment with the mixture, G_1_ and G_2_ are the MF obtained for treatment with the individual chemicals, and C is the MF obtained for the control. A negative IF denotes antagonism, a positive IF denotes synergism, and a zero IF denotes additivity.

The standard error of the mean (SEM) of the IF was calculated as described by Danesi et al. (2012) using Equation 4:


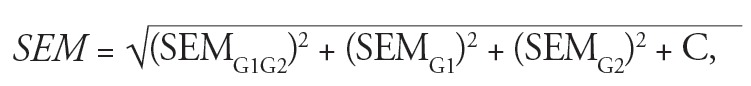
, [4]

where SEM_G1G2_ is the SEM for the mixture.

*Independent action (IA).* Concentration–response relationships for mixtures of compounds are predicted based on concentration–response data for individual mixture components, assuming additivity (Rajapakse et al. 2001). Synergism and antagonism can be defined as deviations from the expected effects, with synergistic mixtures showing higher, and antagonistic mixtures lower, responses than predicted. When the predictions are met, the combined response is additive (Berenbaum 1989). Independent action (IA) describes situations where compounds act on different subsystems, possibly involving different sites and modes of action (Rajapakse et al. 2001). Because the chemicals used in this study have different mechanisms of action [both chemicals are activated by CYP1A1, and PhIP is additionally activated by CYP1A2 (Crofts et al. 1997; IARC 2010; Zhao et al. 1994), and because DNA damage from BaP is a result of ROS and epoxides whereas PhIP induces DNA damage via a nitrenium ion (IARC 2010; Singh et al. 2010)], IA was also used to determine the expected response.

IA can be calculated using Equation 5 as described by Berenbaum (1989):

E(d_a_, d_b_) = E(d_a_) + E(d_b_) – E(d_a_)E(d_b_), [5]

where E(d_a_, d_b_) is the fractional effect of the mixture, and E(d_a_) and E(d_b_) represent the fractional effects of the individual chemicals. In this equation, fractional effect E is used as a substitute for the probability of occurrence of an event and fractional lack of effect (Berenbaum 1989). When applying this model, a maximal effect must be defined (Rajapakse et al. 2001). In the present study, the fractional effect E was the MF, which was expressed as the number of mutants per 10^6^ viable cells; thus, we assumed that the unit of assessment was the cell and that the maximal effect was 10^6^ mutants per 10^6^ cells.

IA was calculated using Equation 6, based on the equation employed by Abendroth et al. (2011):

IA = E_1_ + E_2_ – (E_1_E_2_/10^6^), [6]

where IA is the predicted mixture percent response assuming additivity, E_1_ is the observed percent response for chemical 1 alone, and E_2_ is the observed percent response for chemical 2 alone.

*Statistical analysis.* Statistical significance was determined using a one-way analysis of variance (ANOVA) with Dunnett’s post-test. Statistical significance was defined as *p* < 0.05.

## Results

**Figure 1:**
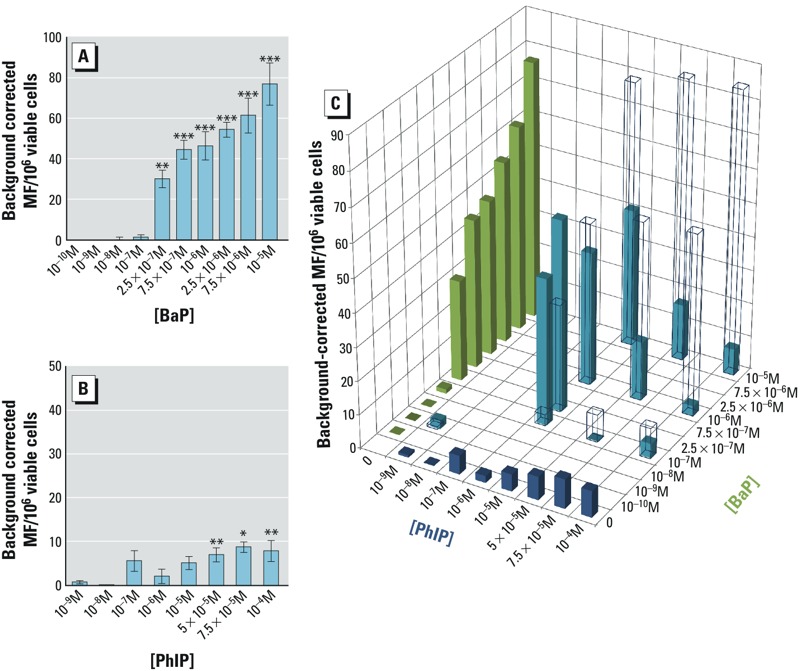
Effects of BaP, PhIP, or BaP/PhIP mixtures on mutant frequency (MF) at the TK locus. Background-corrected MF at the TK locus following a 24-hr treatment with (*A*) BaP, (*B*) PhIP, or (*C*) BaP/PhIP mixtures; in (*C*), open bars represent the predicted MF based on additivity, and solid bars represent the actual MF. The green bars represent the measured MF for PhIP alone, the blue bars represent the measured MF for BaP alone, and the teal bars represent the measured MF for PhIP/BaP mixtures. DMSO negative control average range, 4.9–18.6 MF/10^6^ viable cells; EMS positive control average range, 74.6–125.5 MF/10^6^ viable cells. Historical controls: TK: DMSO, 13.3 ± 9.4 MF/10^6^ viable cells ± standard deviation; EMS (positive control), 99.4 ± 40.4 MF/10^6^ viable cells ± standard deviation; Data are means ± SEM, *n *= 3–12 independent cultures. Significance compared with DMSO control [one-way analysis of variance (ANOVA) with Dunnett’s post-test; *p ≤ 0.05, **p ≤ 0.01, ***p ≤ 0.001].

*TK forward mutation assay for individual chemicals.* The concentrations of BaP and PhIP that were used were chosen to cover a range of values from typical human dietary exposure (< 10^–8^ M) (IARC 2010; Sinha et al. 2005) to high concentrations that induce a high mutant frequency (Felton et al. 2002; Yadollahi-Farsani et al. 1996). BaP produced a statistically significant increase in TK MF from 2.5 × 10^–7^ to 10^–5^ M (Figure 1A), whereas treatment of cells with PhIP required higher doses than those used for BaP (Figure 1B). PhIP has been reported to be a poor mammalian cell mutagen in *in vitro* assays (Knize et al. 2002; Yadollahi-Farsani et al. 1996), requiring doses in the 10^–5^–10^–4^ M range, consistent with the present study.

*Mutant frequency at the TK locus for binary mixtures.* The observed TK MF differed from the expected additive response (based on addition of the MF of individual chemicals and on the IA model). In general, MF was increased for low-concentration mixtures and decreased for high-concentration mixtures relative to the expected response if the MF for individual chemicals was additive. For example, a remarkable statistically significant increase in MF was observed for the combination of 10^–7^ M BaP and 10^–6^ M PhIP (TK MF = 43.6 ± 7.0), whereas these concentrations of BaP and PhIP alone did not significantly increase the MF (TK MF = 1.3 ± 1.2 and 5.6 ± 2.4, respectively) (Figure 1C; Table 1). In contrast, the MF observed for 10^–5^ M BaP combined with 10^–4^ M PhIP (TK MF = 7.9 ± 2.5) was considerably lower than anticipated, given that individually, these concentrations produced significant increases in MF (TK MF = 76.9 ± 10.5 and 7.9 ± 2.4, respectively) (Figure 1C and Table 1). The RTG for the different mixtures did not change significantly from the RTG observed when the same concentrations were tested for the chemicals individually, suggesting no significant toxicity from the individual or combined treatments (Table 1).

*Statistical analysis of the binary mixture data.* Three methods of statistical determination of interaction were employed to assess whether the effects of mixtures of BaP and PhIP were additive, synergistic, or antagonistic. The median effect equation derived from the mass action law principle (Chou 2006) allows quantitative determination of chemical interactions that lead to biological responses. This approach has previously been employed for mixtures where a maximum effect is achievable, (e.g., enzyme inhibition) (Chou and Talalay 1984), but has not, to our knowledge, been applied to mutation data. Here, we define a theoretical maximum effect limit for mutation (i.e., 10^6^ mutants per 10^6^ cells). This assumption is not achievable in practice because mutation at this frequency is incompatible with survival. An alternative method is to use the interaction factor (IF) (Schlesinger et al. 1992), which has recently been applied to genotoxicity data in *Drosophila* (Danesi et al. 2012). Finally, response addition based on independent action (IA), which represents a situation in which compounds act on different subsystems that may involve different sites and modes of action (Rajapakse et al. 2001), was calculated for the mixtures. This method determines outcome based on additivity, and synergism and antagonism can be defined as deviations from the expected effects. The results from all three analyses showed a synergistic interaction for the combination of BaP 10^–7^ M and PhIP 10^–6^ M, and the difference between the observed and predicted joint effect was statistically significant based on IA. In contrast, six combinations of BaP ≥ 10^–6^ M with doses of PhIP ≥ 10^–6^ M were consistently categorized as antagonistic by all three methods, with statistically significant differences between the observed and predicted joint effects based on IA for five of the six combinations (Table 2).

**Table 2:** Analysis of the mutation frequency data at the TK locus for binary mixtures of BaP and PhIP by the median effect equation and the combination index theorum (CI), interaction factor (IF), or independent action (IA).

BaP + PhIP^*a*^	fa^*b*^	CI^*c*^	Mechanism (CI)^*d*^	IF ± SEM^*e*^	Mechanism (IF)^*e*^	Predicted MF with IA^*f*^	Mechanism (IA)^*g*^
10^–9^ + 10^–9^	2.47 ± 2.13	0.02	sS	1.64 ± 3.42	NDAd	0.83	NDAd
10^–7^ + 10^–6^	43.60 ± 11.38	0.007	sS	40.25 ± 7.61	S	3.35***	S
10^–7^ + 5 × 10^–5^	0.10 ± 4.72	3.83 × 10^6^	sA	–8.15 ± 4.53	A	8.25	A
10^–7^ + 10^–4^	4.28 ± 2.81	9.02	sA	–4.89 ± 4.35	NDAd	9.18	NDAd
2.5 × 10^–7^ + 10^–6^	56.81 ± 10.68	0.29	sS	31.44 ± 11.83	S	32.18*	S
10^–6^ + 10^–6^	39.71 ± 4.56	1.25	mA	–8.81 ± 8.74	A	48.52	A
10^–6^ + 5 × 10^–5^	17.50 ± 4.17	3.99	sA	–35.92 ± 8.55	A	53.42***	A
10^–6^ + 10^–4^	2.93 ± 3.14	83.33	sA	–51.42 ± 8.29	A	54.35***	A
10^–5^ + 10^–6^	41.10 ± 5.22	2.42	A	–37.92 ± 12.05	A	79.02***	A
10^–5^ + 5 × 10^–5^	16.85 ± 5.28	8.52	sA	–67.07 ± 12.07	A	83.92***	A
10^–5^ + 10^–4^	7.92 ± 2.49	25.54	sA	–76.93 ± 11.28	A	84.85***	A
Abbreviations: A, antagonism; NDAd, not different from additive; S, synergism. ^***a***^Molar concentration (BaP is shown first); ^***b***^Fraction affected (f_a_) is the background-corrected observed mutation frequency for the combinations/10^6^ viable cells ± SEM; ^***c***^CI = (D_1_/Dx_1_) + (D_2_/Dx_2_); D_1_, D_2_ are the concentrations used in the mixture, and Dx_1_, Dx_2_ are the concentrations of individial chemicals required to achieve the mixture effect; ^***d***^Synergism and antagonism are subdivided into nearly additive (NAd, 0.9–1.1), moderate synergism/antagonism (mS, 0.7–0.90/mA,1.1–1.45), synergism/antagonism (S, 0.3–0.7/ A, 1.45–3.3), and strong synergism/antagonism (sS, < 0.1–0.3/ sA, 3.3 to > 10); ^***e***^IF = G_1_G_2_ – G_1_ – G_2_ + C ± SEM. A negative IF = antagonism (A), positive IF = synergism (S), 0 = not different from additive (NDAd); ^***f***^IA = MF_1_ + MF_2_ – [(MF_1_MF_2_)/10^6^]; MF_1_ and MF_2_ = individual MF, MF_1_MF_2_ = product of individual MFs; ^***g***^Mechanism deduced by comparison of the predicted MF with the actual MF (fraction affected). Observed and predicted MF response compared using a *t*-test with Bonferroni multiple comparisons test (*p ≤ 0.05; ***p ≤ 0.001). Variance surrounding the expected MF was assumed to equal the variance for the observed data (Abendroth et al. 2011).							

**Figure 2:**
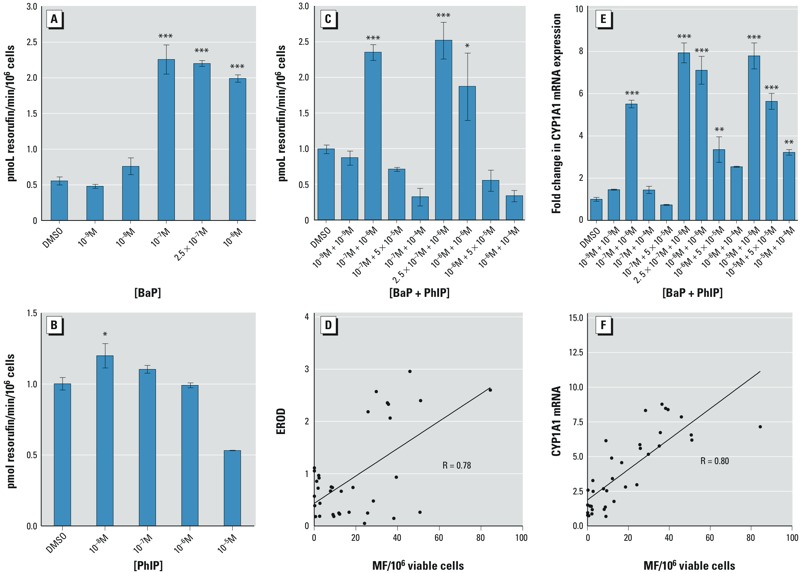
Effects of BaP and PhIP mixtures on CYP1A activity and CYP1A1 expression. For all mixtures, the concentration of BaP is stated first. Ethoxyresorufin-*O*-deethylase (EROD) activity was determined following a 24-hr treatment with (*A*) BaP, (*B*) PhIP, or (*C*) mixtures of BaP and PhIP. (*D*) *CYP1A1* mRNA levels (Q-PCR, normalized to GAPDH) following a 24-hr treatment with mixtures of BaP and PhIP. Correlation of mixture TK MF with (*E*) mixture EROD or (*F*) mixture *CYP1A1* mRNA levels. Data are means ± SEM, *n *= 3. Significance compared with the DMSO control (one-way ANOVA with Dunnett’s post-test; *p ≤ 0.05, **p ≤ 0.01, ***p ≤ 0.001).

*Ethoxyresorufin-*O*-deethylase (EROD) activity.* BaP and PhIP must undergo metabolic activation catalyzed by CYP1A to become genotoxic. EROD, an indicator of CYP1A activity, was measured in cells treated with BaP or PhIP alone or with mixtures of BaP and PhIP. The results showed induction of EROD activity at concentrations ≥ 10^–7^ M BaP (Figure 2A), whereas induction was only observed with 10^–8^ M PhIP (Figure 2B). For the selected combinations, the results showed induction of EROD activity for mixtures of 10^–7^ M BaP and 10^–6^ M PhIP, 2.5 × 10^–7^ M BaP and 10^–6^ M PhIP, and 10^–6^ M BaP and 10^–6^ M PhIP, with no induction observed for other combinations tested (Figure 2C). EROD activity for mixtures was significantly correlated with TK MF (*p* = < 0.0001, *R* = 0.78; Figure 2D).

It should be noted that EROD activity cannot be measured > 10^–5^ M BaP because this is above the Km for CYP1A1 where BaP outcompetes 7-ER for CYP (Crofts et al. 1997).

CYP1A1 *mRNA expression levels.* qRT-PCR performed on cells treated with mixtures of BaP and PhIP showed significant increases of *CYP1A1* mRNA (compared with control) for mixtures with concentrations ≥ 10^–7^ M BaP and 10^–6^ M PhIP. However, at each BaP concentration ≥ 10^–7^ M, the increase in CYP1A1 expression diminished as the PhIP concentration increased (e.g., for mixtures of 10^–5^ M BaP plus PhIP at concentrations of 10^–6^, 10^–5^, and 10^–4^ M) (Figure 2E). The *CYP1A1* mRNA levels were significantly correlated with the TK MF profile (*p* = < 0.0001, R = 0.80; Figure 2F).

*Cell cycle.* To determine whether alterations in cell cycle played a role in the altered MF response that was observed after exposure to combinations of BaP and PhIP, cell cycle status was measured following a 24-hr treatment with selected combinations and after 24-hr and 48-hr recovery phases.

We observed a significant decrease in the number of cells in S phase after 24 hr for the two highest dose combinations [10^–5^ M BaP combined with either 5 × 10^–5^ M (*p* ≤ 0.05) or 10^–4^ M PhIP (*p* ≤ 0.01); Table 3]. There was also a corresponding significant increase in the sub-G1 population, which was significant for all combinations except for 10^–9^ M BaP mixed with 10^–9^ M PhIP (Table 3), a finding that is suggestive of apoptosis.

**Table 3:** Effects of selected combinations of BaP and PhIP on cell cycle distribution assessed by flow cytometry.

BaP + PhIP (M)	24 hr				24 hr post				48 hr post			
	Sub G1	G1	S	G2/M	Sub G1	G1	S	G2/M	Sub G1	G1	S	G2/M
DMSO	4.99 ± 1.30	34.30 ± 2.05	21.93 ± 1.70	24.30 ± 1.31	0.97 ± 0.36	36.20 ± 0.83	30.97 ± 1.68	21.80 ± 0.81	0.11 ± 0.24	43.97 ± 0.91	21.23 ± 0.52	23.00 ± 0.70
10^–9^ + 10^–9^	4.92 ± 0.77	34.77 ± 1.57	19.47 ± 0.59	26.17 ± 0.97	1.27 ± 0.20	35.10 ± 0.46	31.47 ± 1.91	22.90 ± 1.87	1.08 ± 0.39	42.93 ± 1.02	19.77 ± 1.29	22.37 ± 1.23
10^–7^ + 10^–6^	4.72 ± 0.51	35.73 ± 1.34	22.87 ± 0.98	23.80 ± 0.72	2.38 ± 0.07*	37.07 ± 0.90	28.60 ± 2.7	24.97 ± 1.12	1.30 ± 0.52	40.97 ± 0.94	20.80 ± 0.45	23.83 ± 0.99
2.5 × 10^–7^ + 10^–6^	6.65 ± 1.12	33.93 ± 1.77	22.80 ± 1.27	23.00 ± 1.18	2.99 ± 0.50***	36.30 ± 0.90	26.97 ± 1.36	27.07 ± 1.30	1.46 ± 0.24	40.00 ± 2.48	20.77 ± 2.00	24.37 ± 0.43
10^–6^ + 5 × 10^–5^	7.34 ± 1.03	34.30 ± 1.05	18.07 ± 1.19	25.77 ± 0.10	2.45 ± 0.27*	33.50 ± 0.62	28.80 ± 1.03	28.40 ± 0.40*	1.59 ± 0.90	36.60 ± 2.36*	22.27 ± 1.00	25.37 ± 0.88
10^–5^ + 10^–6^	7.41 ± 1.08	33.90 ± 0.67	22.97 ± 1.74	22.57 ± 0.55	3.84 ± 0.16***	32.43 ± 0.58	31.27 ± 2.07	26.70 ± 2.31	6.08 ± 1.31	37.93 ± 1.61	21.57 ± 4.53	24.23 ± 1.51
10^–5^ + 5 × 10^–5^	8.74 ± 1.04	34.47 ± 0.73	15.23 ± 0.55*	25.93 ± 0.53	3.67 ± 0.25***	33.60 ± 1.06	28.80 ± 2.45	29.73 ± 1.79**	2.10 ± 0.17	40.23 ± 2.47	22.57 ± 1.18	24.33 ± 0.07
10^–5^ + 10^–4^	13.00 ± 2.40**	39.73 ± 2.31	14.07 ± 1.24**	22.13 ± 0.44	3.38 ± 0.29***	32.83 ± 1.07	29.57 ± 1.04	30.73 ± 0.77***	1.89 ± 1.08	34.77 ± 1.56**	26.03 ± 0.49	23.93 ± 1.77
Data are presented as the percentage of cells in each phase of the cell cycle ± SEM, *n *= 3. MCL-5 cells were treated for 24 hr, as indicated, then harvested or left in fresh media for a further 24 hr (24 hr post) or 48 hr (48 hr post) after treatment. Dimethylsulfoxide (DMSO) was used as a negative control. Significance compared with the negative control was calculated using one-way ANOVA with Dunnett’s post-test (*p ≤ 0.05, **p ≤ 0.01, ***p ≤ 0.001).												

**Figure 3:**
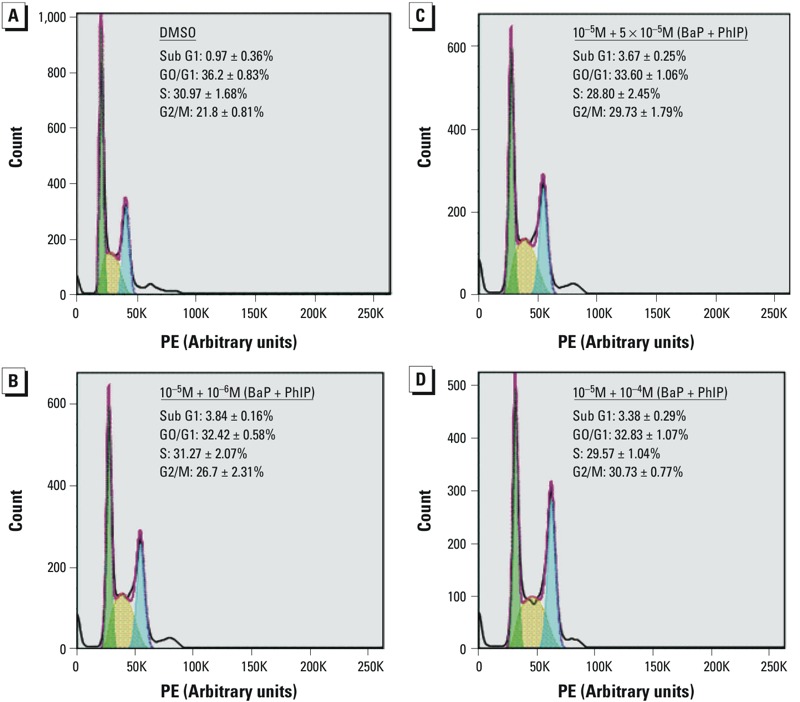
Effects of mixtures of BaP and PhIP on cell cycle. Percentage of cells in each phase of the cell cycle after a 24-hr treatment with (*A*) dimethylsulfoxide (DMSO; negative control) or with (*B*, *C*, *D*) selected combinations of BaP and PhIP followed by a 24-hr recovery period. Assessed by flow cytometry. PE, propidium iodide staining.

Following a 24-hr recovery period, significant increases in the number of cells in sub-G1 were observed for combinations of ≥ 10^–7^ M BaP with 10^–6^ M PhIP, suggestive of apoptosis, and a significant increase in the number of cells in G2/M phase was observed for mixtures of 10^–6^ M BaP with 5 × 10^–5^ M PhIP and of 10^–5^ M BaP with either 5 × 10^–5^ or 10^–4^ M PhIP (Figure 3C,D and Table 3). Accumulation of cells in G2/M phase occurred for mixtures where antagonistic effects were observed and may reflect a block in the cell cycle to allow DNA repair.

Following a 48-hr recovery period, a significant accumulation of cells in G1 phase was observed for the combinations of 10^–6^ M BaP with 5 × 10^–5^ M PhIP and 10^–5^ M BaP with 10^–4^ M PhIP (Table 3). This pattern of cell-cycle phase accumulation is indicative of a population of synchronized cells moving through the cycle after arrest release (Creton et al. 2005; Zhu et al. 2000).

*Expression of mismatch repair proteins MSH2 and MSH6.* PhIP has been linked to induction of G2 arrest and to an increase in levels of MSH6/GTBP (Creton et al. 2005). To determine whether the reduced MF observed at some concentrations of the mixtures was associated with increased DNA repair, levels of mismatch repair proteins MSH2 and MSH6 (hMutSa complex) were measured.

**Figure 4:**
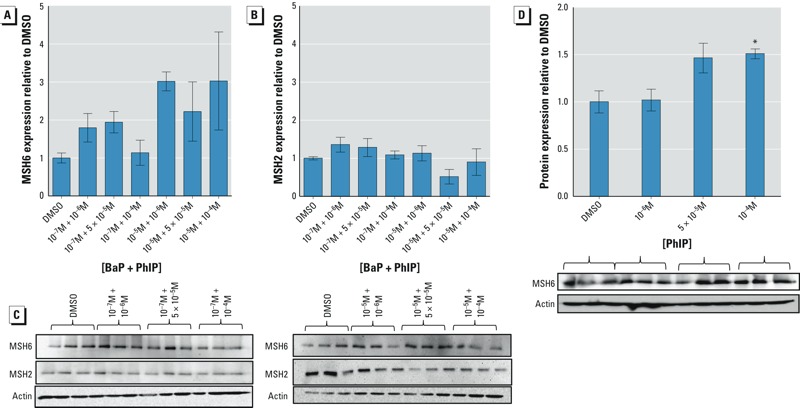
Effects of mixtures of BaP and PhIP, or PhIP alone, on mismatch-repair proteins. Effects of a 24-hr treatment with selected concentrations of mixtures of BaP (stated first) and PhIP on the expression of (*A*) MSH6 or (*B*) MSH2. (*C*) Representative immunoblots showing the abundance of MSH6 and MSH2 proteins. (*D*) Effects of a 24-hr treatment with selected concentrations of PhIP on MSH6 protein expression. The intensity of each protein in DMSO-treated cells was used as a reference after correcting for loading (β-actin). Data are presented as the mean ± SEM, *n *= 3. Significance compared with the DMSO control calculated using one-way ANOVA with Dunnett’s post-test (*p ≤ 0.05).

Following treatment with selected mixtures of BaP and PhIP (10^–7^ M or 10^–5^ M BaP with either 10^–6^ M, 5 × 10^–5^ M, or 10^–4^ M PhIP), MSH6 protein levels were apparently greater for mixtures containing 10^–5^ M BaP than for combinations containing 10^–7^ M BaP (although no combinations tested were statistically significantly different from the control) where antagonistic induction of TK MF was observed (e.g., 10^–5^ M BaP with 10^–6^ M PhIP) (Figure 4A,C). No change in the level of MSH2 was observed (Figure 4B,C).

Because PhIP has been linked with induction of G2 arrest and mismatch repair (Creton et al. 2005; Duc and Leong-Morgenthaler 2004), levels of MSH6 were measured after treating cells with PhIP for 24 hr. A dose-dependent increase in MSH6 protein was observed; however, this increase was only significant at a PhIP concentration of 10^–4^ M (Figure 4D), suggesting that PhIP may be responsible for induction of MSH6 protein by the mixtures.

## Discussion

Eating cooked red meat is strongly correlated with diet-associated cancers, and cooking meat leads to the formation of chemical carcinogens such as BaP and PhIP (Sinha et al. 2005). Many studies have investigated DNA damage caused by individual chemicals, but few have examined the consequences of exposure to chemical mixtures. The present study aimed to examine mixtures of the food-borne genotoxic carcinogens BaP and PhIP at doses that are relevant to human exposure.

The results from the TK mutation assay showed that BaP induced a statistically significant increase in MF at concentrations > 10^–7^ M, whereas PhIP significantly increased MF at concentrations ≥ 5 × 10^–5^ M. In V79 Chinese hamster cells, MF at the HPRT locus was more pronounced in response to PhIP (Yadollahi-Farsani et al. 1996) than was TK MF in MCL-5 cells exposed to the same PhIP cocentrations in the present study. However, V79 cells express a nonfunctional p53 protein (Chaung et al. 1997) and are more susceptible to mutation than MCL-5 cells, which have a functional p53 response (Guest and Parry 1999).

It is noteworthy that 10^–7^ M BaP and 10^–6^ M PhIP did not increase MF with individual exposure (Table 1), whereas in combination, they induced a significant mutation response that was synergistic based on CI, IF, and IA analyses (Table 2). A recent report using the micronucleus assay showed that binary mixtures of chemicals with dissimilar actions at their “no observed genotoxic effect levels” induced a significant increase in micronuclei, supporting our findings (Johnson et al. 2012). In contrast, the combined effect for the combination of 10^–5^ M BaP and 10^–4^ M PhIP was significantly and substantially lower than the effect predicted based on an expectation of additive effects.

Both compounds require metabolic activation by CYP1A1 family enzymes (IARC 2010; Zhao et al. 1994). EROD activity was significantly correlated with the trend for MF, suggesting that CYP1A is required for mutation. In support of this hypothesis, *CYP1A1* mRNA levels were strongly correlated with TK MF. The increase in expression and activity of CYP1A1 was expected because BaP induces CYP1A1 expression via the aryl hydrocarbon receptor (AhR) (Nebert et al. 1993). The correlation of CYP1A1 expression and activity with the observed TK MF suggests that increased activation of the chemicals may represent one reason for the observed synergism. The Kms of BaP and PhIP for human CYP1A1 are 8.8 μM and 5.1 μM, respectively, and the Km of PhIP for human CYP1A2 is 79 μM (Crofts et al. 1997; Schwarz et al. 2001). Thus, for mixtures where MF synergy was observed, the BaP/PhIP concentrations were below the Km of CYP1A1 and CYP1A2, and the enzymes were working with maximum efficiency. Unexpectedly, the induction of *CYP1A1* mRNA and EROD decreased as the concentration of PhIP in the mixture increased, in line with the lower TK MF observed for these mixture concentrations. A possible explanation for the lack of induction of CYP1A1 with increasing PhIP in the mixture is that PhIP is estrogenic and can mediate gene transcription via the estrogen receptor (ER) (Lauber et al. 2004). Aryl hydrocarbon receptor nuclear translator (ARNT) is recruited to estrogen-responsive promoters in the presence of estradiol (Swedenborg and Pongratz 2010); thus, PhIP may be recruiting ARNT to ER, reducing its availability for AhR and CYP1A1 transcription and therefore for CYP1A activity. Although elevation of CYP1A mRNA was observed for high-concentration mixtures (PhIP with 10^–5^ M BaP), the observed increases in TK MF were less than additive. At these high concentrations, access to metabolic enzymes becomes competitive (based on the Kms for BaP and PhIP), thus limiting the formation of DNA-damaging metabolites and resulting in antagonism of MF.

Another possible explanation reflects the cell cycle status of cells. Analysis of the cell cycle 24 hr after treatment with selected combinations of BaP and PhIP revealed significant accumulation of cells in sub-G1 and a block at G2/M, which was dose-dependent with increasing concentrations of PhIP. Treatment with mixtures of 10^–6^ M BaP and 5 × 10^–5^ M PhIP, or 10^–5^ M BaP and either 5 × 10^–5^ M or 10^–4^ M PhIP, led to a significant accumulation of cells in G2/M and an antagonistic effect on TK MF, suggesting that G2/M–phase arrest may play a role in the observed low MF. Although a significant induction of CYP1A1 expression was observed for these combinations, suggestive of increased activation by the chemicals and therefore of increased DNA damage, activation of G2/M–phase arrest may allow damage repair, thereby reducing MF towards baseline levels. Indeed, this G2/M block was not observed 48 hr after treatment, suggesting that the damage had been repaired. The temporal dependency of accumulation of cells at different stages of the cell cycle could reflect release of cells from the initial S-phase block, synchronizing this cell population.

Arrest at G2/M has been reported for these chemicals individually; BaP (10^–5^ M) has been shown to induce G2/M phase arrest following a 48-hr treatment (Drukteinis et al. 2005), and activation of the G2/M checkpoint has been reported 24 hr after PhIP treatment (Duc and Leong-Morgenthaler 2004). Moreover, a recent study showed that complex mixtures of PAHs activated checkpoint kinase 1 (Chk1) (Jarvis et al. 2013), which mediates G2/M phase arrest. G2/M arrest has been linked to mismatch repair for certain types of DNA damage (Aquilina et al. 1999; Duc and Leong-Morgenthaler 2004; Hawn et al. 1995), and the involvement of GTBP/MSH6 in PhIP-induced mutagenesis has previously been reported, with levels of these proteins elevated following PhIP exposure (Creton et al. 2005). In the present study, levels of MSH6 protein appeared to increase more after a 24-hr treatment with mixtures containing 10^–5^ M BaP than for those containing 10^–7^ M BaP. MSH6 forms the MutSα complex with MSH2; this heterodimer binds bulky adducts at the C8 position of guanine produced by aminofluorene (AF) and 2-acetyl-4-aminofluorene (AAF) (Li et al. 1996), and it is believed to be involved in repair of this type of DNA damage. Because PhIP generates bulky adducts at the C8 position of guanine, it is hypothesised that mismatch-repair proteins are also involved in recognizing dG-C8-PhIP adducts (Duc and Leong-Morgenthaler 2004; Glaab et al. 2000). Induction of MSH6 protein supports the induction of cell cycle arrest to repair DNA damage from high-dose mixtures. Interestingly, involvement of DNA repair in nonmonotonic dose responses has been reported in relation to the HPRT assay (Jenkins et al. 2010) and more recently in reference to low-dose “no observed genotoxic effect levels” (Thomas et al. 2013).

## Conclusions

Co-exposure to BaP and PhIP produced mutation responses that differed considerably from those expected based on the IA model of additivity. Combining the demonstrated nonmutagenic dose of 10^–7^ M BaP with the similarly nonmutagenic dose of 10^–6^ M PhIP resulted in a significant increase in TK MF. This effect may be mediated by CYP1A enzymes because EROD activity and *CYP1A1* mRNA were correlated with MF (Figure 2). However, it should be noted that the majority of the tested mixtures led to antagonistic effects. The less-than-additive TK MF observed for high-dose mixtures implies that such combinations are less mutagenic. Our data suggest the involvement of DNA repair, mediated via G2/M-phase arrest, for mixtures containing 10^–5^ M BaP. We hypothesize that in BaP–PhIP mixtures, BaP is the dominant mutagen and makes the greatest contribution to the mutation response. This hypothesis is supported by the increase in *CYP1A1* mRNA levels, which is likely to be BaP-driven because PhIP is a weak inducer of CYP1A1 (Thomas et al. 2006).

The increase in MF for low-concentration mixtures may be of significance when considering the genotoxic potential of food. These concentrations are relevant to human exposure, and as such, our results may have implications for risk assessment because when mixtures are analyzed on the basis of their components, a general assumption is made that interaction effects at low dose levels either do not occur or are small enough to be insignificant to the risk estimate (U.S. EPA 2000). Our data show possible non-monotonic dose responses, and future work investigating DNA adduct formation would help clarify this issue.

When interpreting our observations, the limitations of current *in vitro* mutation assays must be appreciated, and the contribution of metabolism to these processes is a prominent consideration. Although the MCL-5 cell line used in the present study is competent for phase I metabolism of BaP and PhIP, it has limited ability to perform the totality of metabolic reactions that are available in intact mammals. All such *in vitro* mutagenicity models have deficient phase II metabolism, and the majority require added Phase I capability (S9), which limits detoxication, thereby biasing toward a positive mutation response. In this respect, our cell-based system is similar to other *in vitro* mutation assays, and all are likely to overrepresent mutation potential. Thus, detection of MF in *in vitro* mammalian cell systems should be viewed as indicative of mutagenic potential, and further investigation of the mixture concentrations tested herein is required *in vivo* to fully assess the impact of these data on human health and for risk assessment.

Editor’s Note: Equation 4 in the Advance Publication was incorrect. The correct equation is included in this article.
